# Gaps and gains from engaging districts stakeholders for community-based health professions education in Uganda: a qualitative study

**DOI:** 10.1007/s40037-015-0228-2

**Published:** 2015-11-10

**Authors:** Elialilia S. Okello, Joyce Nankumbi, Gad Ndaruhutse Ruzaaza, Evelyn Bakengesa, Joy Gumikiriza, Wilfred Arubaku, Christine Acio, Mary Samantha, Michael Matte

**Affiliations:** 1 Department of Psychiatry, College of Health Sciences, Makerere University, Kampala, Uganda; 2 Department of Nursing, School of Health Sciences, College of Health Sciences, Makerere University, Kampala, Uganda; 3 Department of Community Health, Faculty of Medicine, Mbarara University of Science and Technology, Mbarara, Uganda; 4 College of Health Sciences, Makerere University, Kampala, Uganda; 5 Department of Dental Surgery, Mbarara University of Science and Technology, Mbarara, Uganda; 6 Director, Health and Nutrition, Mbarara Emirates Investments, Mbarara, Uganda; 7 Mbarara University of Science and Technology, Mbarara, Uganda

**Keywords:** Community-based education, Stakeholder, Stakeholder engagement

## Abstract

Community-based education research and service (COBERS) is a brand of community-based education that has been adopted by the Medical Education and Service for All Ugandans consortium. The COBERS programme is aimed at equipping students in health professional education with the knowledge, attitudes and skills required to provide appropriate health care services. For sustainability purposes, the health professional training institutions have made efforts to involve various stakeholders in the implementation of the programme. However, the actual engagement process and outcome of such efforts have not been documented. This paper documents gaps and gains made in engaging district stakeholders for community-based education. Key informant interviews, focus group discussions and document review were used to collect data. Atlas.ti, computer software for qualitative data was used to aid analysis. The analysis revealed that the adopted engagement model has registered some gains including increased awareness among district leaders about potential opportunities offered by COBERS such as boosting of human resources at health facilities, opportunities for professional development for health care workers at health facilities, and establishment of linkages between prospective employees and employers. However, the engagement model left some gaps in terms of knowledge, awareness and ownership of the programme among some sections of stakeholders. The apparent information gap about the programme among district stakeholders, especially the political leadership, may hinder concerted partnership. The findings highlight the need for health professional education institutions to broaden the scope of actively engaged stakeholders with the district level.

## Introduction

Community-based education is a pedagogical model which connects classroom-based work with meaningful community involvement and exchange [1, 2]. Community-based education research and services (COBERS) is a brand of community-based education being implemented by the Medical Education and Services for All Ugandans consortium [3, 4]. COBERS is a hybrid model of community-based education which combines clinical training, service provision and research [5]. In this model, all students enrolled at a university for health professional training are placed under the supervision of one or more community-based preceptors. During placement, the students interact with health workers and district leaders of the host districts. They also interact with patients at health facilities and in the community, learn basic skills and give a helping hand in service provision. In addition, students are required to conduct a research project addressing priority health problems in the community. Given that COBERS activities require the active participation of community-based stakeholders, especially leaders of host districts, it becomes imperative to constantly engage community-based medical education stakeholders outside health professional education institutions [6]. A number of studies have found a clear correlation between the quality of stakeholder relationships and programme performance [7, 8]; sustainable long-term value [9] and programme/institution reputation [10].

Nonetheless, the ability to effectively engage stakeholders begins with a full understanding of stakeholder identity and assigned role, vested interests, and the power to influence the programme. Different stakeholders have different levels of authority (power) over the programme outcome, concerns (interest) regarding the programme and levels of involvement (influence) [11]. It is important that the stakeholder analysis focuses on all these dimensions.

In Uganda, the district administrative structure is divided into two wings: political and technical. The political wing consists of a district council, a policy-making organ, composed of elected councillors headed by a district local council chairperson. The chairperson is supported by an executive committee of a maximum of 5 members and 5 sectoral committees. The five sectoral committees include: finance and planning; health and environment; production, marketing and industry; works and urban planning; and education and sports [12]. The technical wing, on the other hand, is headed by a chief administrative officer and is composed of appointed technical personnel who work in the respective district departments of health, education, roads and transport, finance and planning. The district health service department, headed by a district health officer, is responsible for health service provision in the district.

**Fig. 1:**
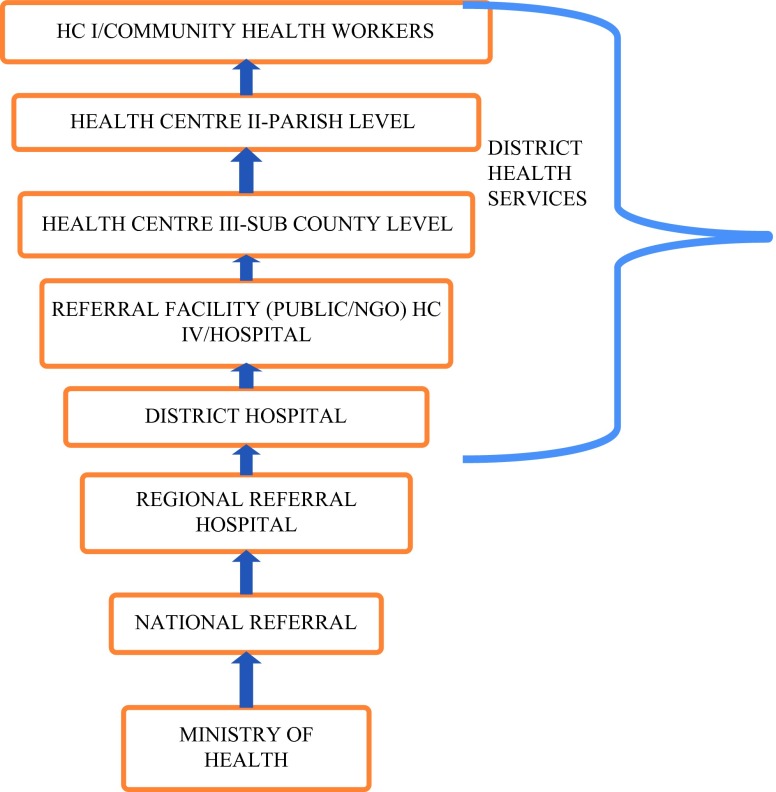
Uganda’s health care system administrative structure.

The Uganda health care system is aligned with the administrative structure with eight [8] service provision tiers (Fig. 1).

Five of the eight tiers are managed by the district health services department as indicated in Fig. 1. The tiers include: health centre I at village level, health centre II at parish level, health centre III at the sub-county level, health centre IV and the district hospitals. COBERS training utilizes the health provision tiers under the district health service department.

The institutions in the Medical Education and Service for All Ugandans consortium adopted *the bridging engagement approach* which involves selecting and prioritizing stakeholders [13]. The approach engenders opportunities for stakeholders to actively participate in the programme. However, the outcome of this engagement and perception of stakeholders regarding the programme has not been documented. This paper documents gaps and gains made in engaging district stakeholders for community-based education programme in Uganda.

## Methods

### Study approach

The study was conducted with the goal to improve the understanding of COBERS and how it is employed, and to improve the environment in which COBERS is implemented [14]. We used *contextual action research*, also referred to as an action learning approach. The approach entails reconstituting the structural relations among actors in a social environment, and recognizes the importance of involving all affected parties and stakeholders as each participant understands the working of the whole. It stresses that participants act as programme designers and co-implementers [15].

### Design, sites and participants

The aim of the study was to identify the gaps and gains made in engaging district stakeholders for the community-based education programme in Uganda. A combination of methods was used to collect data including key informant interviews to elucidate the COBERS engagement process within the districts and focus group discussions to assess participants’ knowledge and awareness of COBERS. Interviews and focus group discussions were tape recorded and transcribed verbatim. In order to corroborate the findings from the interviews and focus group discussions, the minutes of meetings, curriculum documents, proceedings of workshops, and memoranda of understanding between health professional education institutions and key stakeholders in community-based education were reviewed. All the documents relevant to COBERS were accessed, assessed and compiled. A data collection form to summarize the documents was created. The items of the form included: type of document and information to be extracted. The information from the documents was then summarized. For interviews and focus group discussions, purposive sampling methods were used to select districts and study participants. In order to obtain stakeholder perspectives from all the regions of Uganda, two districts were selected from each of the four regions to make a total of eight districts for the study. Participant eligibility depended on the leadership role one played at the district local government. Eight focus group discussions were conducted with key district leaders while key informant interviews were conducted with individuals in charge of the COBERS programme in the health professional education institutions. Interview and focus group discussion guides were used to explore how various stakeholders in the district were engaged in COBERS and also how information was shared between health professional education institutions, district contact persons and district leadership. To assess level of awareness and knowledge among the district stakeholders, participants were asked what COBERS meant and what they perceived to be potential gains that would come with active participation of districts in COBERS activities.

### Data analysis

Thematic analysis was utilized to analyze the data. We used a workshop approach to data analysis, whereby each author independently read each transcript several times to get familiar with the depth and breadth of the data content, identified key words and came up with tentative codes [16, 17]. The tentative codes were shared and discussed among the authors to reach consensus on an appropriate code list. A code book with a comprehensive code list and corresponding code definitions was developed. In line with the study objective and information emerging from the data, an inductive analytic approach was used [18], whereby interview questions and themes extracted from the reviewed documents constituted pre-determined themes [17]. Respondents’ answers to each question were coded using Atlas.ti [19]. After coding, data segments were retrieved and patterns in codes for each question across all respondents were identified. A descriptive summary of the memos was then produced. Representative quotes were selected to illustrate the most salient points. Further analysis of the descriptive summaries was performed, taking an interpretative stance to explore the underlying ideas behind the semantic content of the data [16]. Interpretative analysis was informed by the study objective, as well as the COBERS programme documents including stakeholders memoranda of understanding and minutes of meetings between officials from health professional education institutions and community-based stakeholders [18].

### Ethical considerations

Ethical approval was received from the Makerere University, School of Medicine Research and Ethics Review Committee and the Uganda National Council of Science and Technology. Permission was also sought from leaders of the participating districts. Prior to each group discussion a consent form was read out to the participants who were given a chance to ask questions before signing the consent form. Issues of anonymity and confidentiality were emphasized to all the participants.

## Results

The following themes emerged from the data during the analysis: (i) district stakeholder engagement activities; (ii) stakeholder engagement outcomes (level of knowledge and awareness about COBERS); and (iii) stakeholder perception about opportunities offered by COBERS. These themes are described and discussed below.

### Stakeholder identification and analysis

Data from document review and key informant interviews indicated that training institutions utilized the power, interest and influence model to identify, analyze and prioritize stakeholders. The analysis focused on identifying stakeholders with power and interest to influence not only the implementation of COBERS but also the outcome of the programme. Based on the analysis, the district local council chairperson who heads the district council, the policy-making organ and the district chief administrative officer who oversees the implementation of policy and budget were identified as key stakeholders with both power and influence. Similarly the district health officer who heads the health service department was identified as an important stakeholder. This was due to the power vested in the officer as head of the district health service department and the interest in health service provision in the district, as illustrated by the following quotation:

*‘The district health officer was involved because the training of health professionals is more related to his office. The chief administrative officer is involved in the implementation of the policies and budget. The district local council chairperson is the head of the policy-making organ of the district’.* (Representative, Health Professional Education Institutions)

In the Ugandan health service structure, the district health officers oversee and supervise all health service units within the district from which the training sites are selected. Chief administrative officers and district local council chairpersons, on the other hand, were considered to have the power and influence to allocate the needed material and human resources to COBERS. Whereas the health professional education institutions made initial contacts with the districts through the respective district health officers, the memoranda of understanding were signed by respective chief administrative officers on behalf of the districts.

The main engagement activities consisted of consultations, dialogues and sharing information about COBERS with the identified district stakeholders.

*‘Shared information included: the meaning of COBERS; justification for COBERS, its potential role in retaining human resources for health as well as the role of various stakeholders’.* (Representative, Health Professional Education Institutions)

Channels of information sharing between the health professional education institutions and the districts participating in COBERS included the following: health professional education institutions sending a representative to the annual meetings of the Uganda Local Government Association; health professional education institutions organizing and facilitating district leaders regional meetings; one-on-one meetings with key individuals in the districts; formal introduction letters given to students at the beginning of attachment; and students holding briefing and debriefing meetings with district leaders.

*‘We make courtesy calls to their respective offices; invite them to participate in meetings. For instance recently we had a meeting for chief administrative officers and local council chairpersons from Western Uganda where we invited about 26 districts participating in COBERS activities. Other meetings were also held in 2012. We also participate in the local government meetings held annually’.* (Representative, Health Professional Education Institutions)

There were also collaborative activities related to improving the training sites. For example, districts provided space for accommodation and the universities would make the necessary furnishing and renovation; and the universities engaged preceptors in the training activities to serve as site tutors.

*‘But they had also trained a supervisor who was from the community who would supervise these people’.* (Technical leader, Eastern Region)

The most formal engagement was signing of memoranda of understanding between district authorities and some health professional education institutions, which stipulated partners’ roles and responsibilities.

*‘We have signed memoranda of understanding with several districts where we send students for community-based education service and research. The memorandum is usually signed by the chief administrative officer on behalf of the district and it clearly spells out the roles and responsibilities of each partner’.* (Representative, Health Professional Education Institutions)

### Awareness and knowledge about COBERS

Awareness and knowledge about COBERs varied both across and within districts. Some participants were well informed about community-based education and were also aware of the timing of the students’ placements and the activities that the students were engaged in during placement. Yet, others had very limited or no knowledge about the community placement programme. Overall the technical arm of the district local government, which comprised the chief administrative officer, district health officer, and health facility representatives, was well informed and aware of the COBER’S programme. They were also informed about the different activities such as health education, immunization, and research that the students were involved in during their placement in the districts, as illustrated by the selected quotes below:

*‘Normally the district health officer’s office is informed when the students are coming. A letter is sent which is written to the district health officer’s office informing them of the students who will be attending the programme’.* (Technical leader, Northern Region)

The political leaders were generally less informed about the programme; some thought it was a non-governmental organization or association, while others thought it was a community development project. A few of the leaders had never heard about the programme.

*‘…. but we didn’t know; when you started talking about COBERS I said to myself now this is a new association or something they have just formed’.* (Political leader, Northern Region)

*‘I think that would be best described by the district health officer because earlier on we said that some of us are hearing about COBERS for the very first time’.* (Political leader, Eastern Region)

From the perspective of district leaders, the variation in awareness and knowledge about COBERS could be attributed to the engagement approach used by health professional education institutions coupled by the poor information flow between the technical and political arms of local government. Participants noted that health professional education institutions selected and prioritized stakeholders based on the reality of limited human and financial resources to fully engage each individual stakeholder*.* The assumption was that the information shared with the priority stakeholders would trickle to other stakeholders within the districts through various communication channels. However, this did not happen in most districts. Furthermore, it was noted that leadership structure may have contributed to the information gap about the programme. More often than not, there were communication gaps between political and technical wings of the district leadership, as illustrated by the quotation below:

*‘As said earlier, as leaders we were not aware about this. The district health officer was aware about it, but it was not brought to the attention of the head of the leadership’.* (Political leader, Northern Region)

According to the respondents, the information gap was impeding a sense of ownership among district leaders, yet it was crucial for full participation in the training of health professionals.

*‘When top district leadership is informed, the matter will automatically move to council. You know what it means; when it is owned by council, we can fully participate’.* (Political leader, Western Region)

## Opportunities for participation

During the discussions, the participants reported that COBERS presented various opportunities both to the communities and the health services sector in the districts. They included: boosting human resources, promotion of continuous health professional education for health workers in health facilities, and improved time management at health facilities. Participants also cited the potential for students’ research to inform health-related decision making and investments, and community-based education’s potential to link employers with potential future employees.

### Human resource boost

For most respondents, the students participating in the COBERS programme were seen as a boost to human resources. Students attached to health facilities participated in the day-to-day running of health facilities, including outreach programmes. They took part in activities such as screening patients, patient education, weighing children, measuring temperatures and immunization, depending on the student’s year of study. According to the respondents, participation of students in health service provision in these human resource constrained settings lead to a reduction in patient waiting times.

*‘When they are there, certainly the work force is increased. We have additional hands; medical students have particularly been very active in our outpatient department and they clear the lines faster and the waiting time lessens’.* (Technical leader, Western Region)

*‘They give an extra hand not only at the health facility but also in the community – especially the community health activities such as immunization and other community outreaches. They are very helpful, and they try to foster our human resources’.* (Technical leader, Eastern Region)

Some respondents observed that the presence of additional student hands had a positive effect on the services offered at health facilities. The presence of a full team at the health facility provides psychological reassurance to patients seeking services.

*‘I think they also improve the quality of care to the patients in the health centres because psychologically when you see a team of people working together it also improves the quality of care and uplifts the patient’s spirit knowing that all these people are trying to help you improve’.* (Technical leader Northern Region)

Similarly, the presence of students in the community was reported to have improved sanitation and hygiene, knowledge on nutrition, and health-seeking behaviours of households in the community in which they work.

In some districts, it was noted that the presence of students in the community was in a way helping to improve the image of health facilities as the students were able to communicate and create a trusting relationship between service users and providers.

*‘At a community level, when these students move into the communities and they use friendly and simpler language, it encourages openness. Some people are afraid to go to the hospital and might not be open about their health problems. But at that level, when you meet them in their communities and you are friendly and communicate in simple language, then they open up and you are able to help them’.* (Political leader, Western Region)

### Promoting health workers skills

District leaders noted that the presence of students motivates health workers to update their own health professional skills in order to be able to impart appropriate knowledge and skills to the students they are assigned to mentor. Through this interaction with students, health workers get access to some up-to-date professional knowledge.

*‘When you get students they can even give you more knowledge on some of the conditions that we normally do not talk about. Sometimes, health workers are forced to update their medical knowledge’.* (Technical leader, Western Region)

The presence of students provided opportunities for preceptors or site tutors to get training on leadership and management, teaching and mentoring skills. Site tutors supervise students’ activities and provide mentorship to students attached to the health facility. To be able to actively participate in the process, they received training to equip them with the necessary skills.

*‘Learning, and knowledge, the institutions normally invite us for training on management and leadership skills and they also give us certificate’.* (Technical leader, Western Region)

### Student research

For some respondents, the research conducted by students during attachment provided the much needed evidence to inform district-level decision making and resource allocation for health services. For example, students’ research in one of the communities helped to identify malnutrition as a major health problem affecting the community. Such information guided subsequent decisions and actions for the public health programme.

*‘Every time the students do research during their attachment, research being what it is, they generate knowledge. At the end of the research, they come to know how best to handle the deficiencies in the community, for example nutrition. Like we were not aware that malnutrition was a serious problem until it was documented by a group of students attached to our health facility’.* (Technical leader, Eastern Region)

### Linking employers to employees

According to respondents, community-based education links the potential employer (local government) and potential employees (students). During the attachment, students are able to appreciate the environments which they could consider for future employment. They also get an opportunity to interact with the district leaders. In addition, the attachment provides an opportunity for cultural exchange, which is important for cultural sensitivity and competence during future professional practice.

*‘I think this it is a good programme bringing the training of the students closer to the ultimate health consumer. We get to know how health professionals are trained and their potential to provide services right from the time of training. The programme provides an opportunity for us in the district to interact with students and interest them in joining us in the district’.* (Political leader, Western Region)

*‘Some of the students who have come to our district were from different parts of the country. This is a good thing because students get exposed to other cultures’.* (Technical leader, Northern Region)

## Discussion

This paper provides an overview of the efforts by health professional training institutions to engage district stakeholders in students’ training and a qualitative assessment of the outcomes of such efforts, including stakeholder views about potential opportunities for participating in a community-based education programme. The findings show that training institutions have made efforts to identify key stakeholders in the districts and engage them through various activities. The engagement process has produced promising results, but there are also gaps in terms of information related to COBERS and the role district stakeholders are expected to play in the implementation of COBERS.

### Engagement process

Health professional education institutions adopted the power, influence and interest criteria to prioritize stakeholders at district level. However, use of such an approach seemingly left some of the potential stakeholders in the district without sufficient information to appropriately influence the implementation of COBERS. The political leaders who are charged with policy making and budget allocation were not well informed about the COBERS programme and therefore did not fully contribute to programme implementation. Yet, evidence shows that a more inclusive engagement process has better outcomes. Some studies have documented prioritization of stakeholders as an important strategy for efficient use of resources [20–22]. It is therefore recommended that even stakeholders with minimum power, interest and influence may need to be fully informed about the programme to ensure they do not oppose or sabotage the programme activities due to a lack of information or feeling of being side-lined [21, 23–25].

### Information gaps

Despite the efforts made to engage and share information with key stakeholders, knowledge about COBERS was varied across leaders, within districts and across districts with some leaders more aware and knowledgeable than others. The contrast was very clear between the political and technical stakeholders of the districts. The district health departments headed by district health officers were more informed than other departments in the districts. The policy makers (the district local council chairpersons and district councillors) had the least information about COBERS.

A review of documents related to COBERS implementation suggests that efforts to engage stakeholders ranged from consultative meetings to formal signing of memoranda of understanding between the training institutions and districts. However, the objective(s) of the memoranda of understanding were seemingly unclear to the district stakeholders which left them detached from the programme. The information gaps among the policy makers at district level leaves a potential synergy between training institutions and the district local government unexplored and untapped. Evidence shows that districts where policy makers were actively involved in COBERS do provide students with accommodation and transport during COBERS placement [6]

The literature shows that the level of stakeholder involvement in partnership plays a key role in determining the amount of synergy that a partnership can create. However, if these participants are not involved in a way that makes it possible for them to contribute their knowledge, resources, and skills, partnership cannot create effective synergy. In addition, the ability to create synergy is influenced by the type of partnership. Two types of partnerships are identified in literature, the *lead agency* and the *community engagement* models [26]. These two models vary considerably in their ability to create synergy. The *lead agency* model refers to a partnership established to help a partner participate in a predetermined programme. The model has limited capacity to create effective synergy for it allows limited stakeholder involvement and participation. In the *community engagement* model, by contrast, stakeholders work together in developing plans and taking collective action [27–30]. Such an interaction has the potential to create new ideas and strategies together, which in turn creates sense of ownership among the partners.

### Opportunities presented by COBERS

Participants pointed to the fact that COBERS presented various opportunities both to the communities and the health service sector in the districts. The presence of students at health facilities and communities surrounding the health facilities boosted the much needed human resource for health. It is well established in the literature that the community-based education programme has various benefits for the community and preceptors [31–33]. The research part of COBERS has created a pool of information that the districts could use for more effective planning, prioritization and delivery of health services. The programme provided an opportunity for potential employers (local governments) and potential employees (health professional trainees) to interact and appreciate each other for informed choices. Similarly, the background mix of students sent to COBERS sites across the regions provided an enriched learning environment for both health workers and students and may help in fostering the right attitudes for working in ‘*hard-to-reach’* areas.

## Conclusion

Health professional education institutions have made efforts to identify and engage key stakeholders at district levels. As a result, stakeholders have been able to recognize several potential opportunities for participating in community-based health professional education. However, there are apparent gaps in terms of information and sense of ownership of COBERS among district stakeholders, especially the political leadership, which may hinder effective partnership as envisaged by the training institutions. Consequently, the training institutions may need to consider changing their approach to information dissemination from contact points to a wide sensitization approach and consider using the identified opportunities to consolidate the partnership with districts.

## Essentials

- Stakeholder engagement is important when implementing a community-based education programme.
- Stakeholder engagement allows stakeholders to delineate their roles in the implementation of the programme for active participation.
- The model of stakeholder engagement influences the level of stakeholder participation.
- The full potential of community-based education, including possible opportunities for stakeholders, can be realized when stakeholders are fully engaged.
